# MicroRNA target prediction using thermodynamic and sequence curves

**DOI:** 10.1186/s12864-015-1933-2

**Published:** 2015-11-25

**Authors:** Asish Ghoshal, Raghavendran Shankar, Saurabh Bagchi, Ananth Grama, Somali Chaterji

**Affiliations:** Department of Computer Science, Purdue University, West Lafayette, IN, 47907 USA; School of Electrical and Computer Engineering, Purdue University, West Lafayette, IN, 47907 USA

## Abstract

**Background:**

MicroRNAs (miRNAs) are small regulatory RNA that mediate RNA interference by binding to various mRNA target regions. There have been several computational methods for the identification of target mRNAs for miRNAs. However, these have considered all contributory features as scalar representations, primarily, as thermodynamic or sequence-based features. Further, a majority of these methods solely target canonical sites, which are sites with “seed” complementarity. Here, we present a machine-learning classification scheme, titled *Avishkar*, which captures the spatial profile of miRNA-mRNA interactions via smooth B-spline curves, separately for various input features, such as thermodynamic and sequence features. Further, we use a principled approach to uniformly model canonical *and* non-canonical seed matches, using a novel seed enrichment metric.

**Results:**

We demonstrate that large number of seed-match patterns have high enrichment values, conserved across species, and that majority of miRNA binding sites involve non-canonical matches, corroborating recent findings. Using spatial curves and popular categorical features, such as target site length and location, we train a linear SVM model, utilizing experimental CLIP-seq data. Our model significantly outperforms all established methods, for *both* canonical and non-canonical sites. We achieve this while using a much larger candidate miRNA-mRNA interaction set than prior work.

**Conclusions:**

We have developed an efficient SVM-based model for miRNA target prediction using recent CLIP-seq data, demonstrating superior performance, evaluated using ROC curves, specifically about 20 % better than the state-of-the-art, for different species (human or mouse), or different target types (canonical or non-canonical). To the best of our knowledge we provide the first distributed framework for microRNA target prediction based on Apache Hadoop and Spark.

**Availability:**

All source code and data is publicly available at https://bitbucket.org/cellsandmachines/avishkar.

## Background

MicroRNAs (miRNAs) are short 20–24 nucleotide (nt), endogenous RNAs that modulate gene regulatory pathways [1, 2] and form the most widely studied class of non-coding RNAs (ncRNAs). miRNAs mediate RNA interference (RNAi) by targeting the 3’ UTR of the mRNA, or in some cases, other mRNA regions, such as the mRNA’s coding sequence (CDS) or its 5’ UTR [3]. Following their biogenesis, miRNAs complex with Argonaute (AGO) proteins, which are the catalytic components of the RNA-induced silencing complex (RISC) [4]. This miRNA-RISC complex then targets its cognate mRNA fragment. These interactions result in mRNA repression, destabilization, or, in more complex ways, contour the gene expression landscape [5, 6]. There are over two thousand miRNAs that have been annotated in humans [7], displaying many-to-many associations with mRNA targets. Such associations are speculated to be controlling a vast majority of mammalian genes [8], involving all cellular pathways, from development to pluripotency to oncogenesis [9–14].

Notwithstanding the biological importance of miRNAs, determining their targets with high accuracy and exhaustively has remained elusive, with *in-silico* predictions plagued by high false-positive and false-negative rates [15]. This is due in many ways to the small size of miRNAs, which requires as few as 6 base pairs of complementarity for functional miRNA targeting, as well as the diverse miRNA targetome [16]. As a machine learning task, the problem of miRNA target prediction is that of link prediction in a bipartite graph, where vertices in one set represent all possible target regions across all mRNAs while vertices in the other set represent miRNAs. We can either predict if an edge exists (1/0) between a pair of vertices representing an mRNA region and a miRNA (classification), or we can predict the strength of the association i.e., edge weights (regression). In this paper, we focus on the classification problem of whether a miRNA targets an mRNA region.

CLIP-seq, crosslinking via immunoprecipitation followed by high-throughput sequencing, an elegant albeit lengthy biochemical procedure, is a state-of-the-art-player in developing genome-scale regulatory insights [17–19]. The technology allows target mRNAs to be identified within a small window of resolution, beyond which, statistical models are needed to exactly localize the MRE, that is, the miRNA recognition element or the binding site. This is true even for recent CLIP-seq variants [19], in order to account for background noise and sequencing artifacts [20, 21]. Further, CLIP-seq has the advantage of profiling the native miRNA levels, as opposed to supra-physiological levels obtained via miRNA transfection experiments [22], the latter being better suited for developing small-interfering RNA (siRNA)-based therapeutics [23, 24].

While CLIP-seq can identify miRNAs and targets that form a part of the RISC complex, it cannot decipher *which* miRNA forms a heteroduplex with *which* targets. CLASH is an initial attempt in experimentally solving this problem [25]. Several computational methods have been developed to decipher the specifics of miRNA-mRNA interactions captured by CLIP-seq [26–31]. These methods have contributed to understanding the diverse nature of interactions between miRNA and mRNA. The evolving knowledgebase has further supported the paradigm switch, wherein it is now widely appreciated that the perfect complementarity between the miRNA seed and the mRNA 3’ UTR is neither necessary nor sufficient for miRNA regulation.

**Our contribution** In this paper, we seek to leverage this ability of the CLIP-seq technology to capture endogenous MREs to develop a unified method to understand the signatures of miRNA-mRNA heteroduplexes. Our method applies equally to standard, canonical seed matches, and non-standard, non-canonical seedless matches^1^. Specifically, in our system, which we call *Avishkar*^2^, we use smooth B-spline, thermodynamic curves and sequence curves for adenosine-uracil (AU) content, in order to extract enriched interaction features from the experimentally CLIPed (i.e., immunoprecipitated) regions.

Our main contributions through this work can be summarized as follows:

1. We develop an efficient Support Vector Machine (SVM)-based classifier to identify the positive miRNA-mRNA interactions. Our classifier produces significantly better ROC curves than all prior work [26–28, 32, 33] when evaluated on CLIP-seq data, while also providing insights on which features are discriminating, and in which direction, that is, positive or negative interactions. Our Area-Under-the-Curve (AUC) values for the ROC curves for both human and mouse datasets are greater than that of all prior works, quantitatively 19.7 % and 22.0 % better for human (seed and seedless respectively) and 15.0 % and 22.8 % better for mouse (seed and seedless respectively).
  The classification performance of our model in inter-species validations while being slightly worse compared to intra-species validations, is still able to beat all prior methods. Our improved performance (in terms of true-positive and false-positive rates) over all prior work arises from a combination of multiple factors, with the total benefit being greater than the sum of the constituents. The contributory factors are the use of an extensive set of features, converting noisy data points into smooth curves, converting the categorical feature of seed or seedless match into a numerical feature and treating both under one unified umbrella, and a careful consideration of the spatial nature of the miRNA-mRNA binding process into our classification scheme. Our candidate dataset of miRNA-mRNA interactions is the largest among other computational approaches, which we achieve by employing the least strict filtering criteria on the original CLIP-seq data. Finally, our method is able to predict significantly more non-canonical sites that are present within CLIPed regions than prior computational approaches.
2. We characterize thermodynamic and sequence scores as “curves” and demonstrate how the shape of the curves discriminates between positive and negative miRNA-mRNA interactions. We compute curves at two levels of granularity for each of the thermodynamic and sequence features—curves centered at the target site (we refer to them as “site curves”) and curves computed at a finer granularity and centered at the mRNA seed-matched region (we call them “seed curves”). We demonstrate that a sum of 20 basis-splines (B-splines), each of degree 3, gives us satisfactory curve-fitting. Our use of B-splines enables us to fit relatively smooth curves over high dimensional, noisy data—the scalar data points for thermodynamic and sequence scores.
3. We develop and incorporate in our model a novel metric called *seed enrichment* that captures all patterns of seed matches, including multiple mismatches, GU wobbles (sequence-based imperfections), and long bulges (architectural imperfections), in forming the miRNA-mRNA heteroduplex. By doing so, we are able to adopt a unified approach toward modeling canonical and non-canonical heteroduplexes. This creates a numerical feature that makes it easier for our ML classifier (and other ML-based approaches) to use this feature for classification. We also demonstrate that a whole gamut of non-canonical seed matches, involving bulges on the mRNA, are enriched in the set of positive miRNA-mRNA interactions, seen in both human and mouse-derived data. In fact, the proportion of non-canonical matches is higher than that of canonical matches. This category of matches had been missed in much of prior work, e.g., [32, 33].

**Importance of seed*and* seedless matches** Early studies on miRNA target recognition revealed near-perfect (contiguous) and conserved, Watson-Crick complementarity at the 5’ miRNA end, which was called the “seed region”. The seed is a 6–8 nt substring within the first 8 nucleotides, starting from the 5’ miRNA end. Typically, positions 2–7 from the 5’ end are considered to be the primary (canonical) determinant of target specificity [34–37]. However, given the large number of random occurrences of any given hexamer in 3’ UTRs, a canonical “seed” match by itself is a poor predictor of miRNA-based regulation [38]. To complicate matters, non-canonical interactions involving “seedless sites”, where the interactions are not nucleated by perfectly complementary miRNA seed regions and yet effectively downregulate gene expression, have been described [39–45]. Popular sequence alignment tools such as BLAST cannot align short sequences with specific bulges or mismatch configurations [46]. Taken together, computational methods for miRNA target prediction have traditionally focused on canonical (seed-based) matches. Along the same lines, interactions with the 3’ UTR mRNA target region have been primarily modeled, as opposed to the 5’ UTR, or CDS, or non-coding mRNA regions. In our work, we remove these two restrictions and find seedless matches (in addition to the seed matches) throughout the gene regions^3^.

### Related work

Among non-canonical prediction methods, mirSVR [26] allows for a single GU wobble or a mismatch in the 6-mer seed region. For encoding the seed match pattern, mirSVR uses an 8-bit long vector, with “1” representing a match and “0” representing a mismatch and then uses the bit-vector as a feature in their Support Vector Regression (SVR) model. Recent methods have expanded the target search to other genic regions, such as, to the 5’ UTR and coding sequence (CDS) [27, 47]. In this bracket, Liu *et al.* generate predictions for sites involving non-canonical (seedless) matches. However, they do not take into consideration the type of non-canonicality for the examined seedless sites. Instead, they use thermodynamic and mRNA sequence features (e.g., local AU content) to generate predictions for the seedless sites. In doing so, they miss out on potential signal from the non-canonical seedless match patterns that our findings indicate as enriched in the identified functional miRNA-mRNA interactions. One possible reason for this, as pointed out by Xu *et al.* [48], is the difficulty in incorporating the large numbers of possible patterns of insertions and deletions in the mRNA seed-matched region for different non-canonical seedless match patterns. Computational methods have also exclusively relied on thermodynamic features, such as the stability of the miRNA-mRNA heteroduplex and the accessibility of the mRNA target region to identify functional miRNA binding sites. For example, Xu *et al.* [48] only use binding energy and accessibility to predict functional miRNA target sites.

Another method, MIRZA [28] develops a rigorous biophysical model via parameterizing the alignment between a miRNA and an mRNA segment, interpreted as the binding energy between the two, and optimized using CLIP-seq data. While MIRZA uses a novel model to incorporate canonical and non-canonical matches in a unified manner, it does not take into account secondary mRNA structures (the spatial configuration) in developing their energy model—mRNA secondary structures can potentially limit the target site accessibility to the docking miRNA-RISC complex and therefore plays an important role in miRNA target recognition [32]. Further, these approaches compute various thermodynamic scores only at the target site region to summarize the thermodynamics of the miRNA-mRNA interaction. For example, Xu *et al.* [48] observe that a certain normalized measure of site accessibility, has a characteristic pattern around the target site region. Yet, they do not exploit this observation in their model. In contrast, while Liu *et al.* [27] compute site accessibility in the target region’s vicinity in discrete chunks of 5, 10, 15, 20, 25, and 30 nt around the target site region, they report only the accessibility computed at the target site region as an important predictor of functional miRNA targets, failing to capture the mRNA secondary structures around the target site, which define its structural accessibility. Identifying this, prompted us to characterize binding energy and accessibility as curves to model the spatial profile of the miRNA-mRNA interaction.

## Results and Discussion

This Section is segmented into four Sub-Sections. In the first, titled “Approach” we describe our overall solution approach. Next, in the Sub-Section titled “Results”, we present our experimental results and draw our observations from them. Third, we compare our performance *vis-à-vis* competition. Finally, we discuss some salient points in developing our model and present potential threats to the validity of our approach.

### Approach

While CLIP-seq datasets identify short mRNA regions that are functional AGO-mRNA interaction sites [18, 49, 50], additional bioinformatic analysis is needed to identify the miRNA-mRNA binding sites. In order to identify miRNAs that might target those AGO-crosslinked regions, we followed the same general approach as previous methods [26, 27, 32, 47]. The idea is to first generate a candidate set of mRNA binding sites for a list of mRNAs and miRNAs by enforcing a minimum threshold on the alignment score^4^ or the minimum free energy of hybridization (*ΔG*) of the miRNA and mRNA and/or using seed-match constraints. In the next step, different methods use varied approaches to identify true miRNA target sites within the generated candidate set of miRNA-mRNA interactions. Previous works have used various criteria to generate the candidate set of mRNA target sites. For example, in mirSVR [26], the authors use the miRanda algorithm [51] to generate the initial candidate set. The miRanda algorithm computes an optimal local alignment of the miRNA with an mRNA sequence, by using various parameters for the overall alignment score, gap opening, and gap extension penalties. The authors generate candidate sites involving canonical seed matches, which they define as “sites that contain minimally a 6-mer perfect match, spanning miRNA positions 2 to 7”, and non-canonical seed matches. However, for the latter, they only allow a single G:U wobble or a single mismatch in the seed region. In [27], Liu *et al.* use two criteria for generating the candidate set. First, they use the RNAhybrid program [52] to generate candidate sites by enforcing a threshold of –15 kcal/mol on the thermodynamic binding energy (*ΔG*). Second, they constrain the seed match alignment, without constraining the binding energy for the match, to belong to one of the five seed classes of miRNA seeds, as defined in [4]. It is easy to see that by starting out with a more restricted set of candidate target sites, a method can achieve a higher true-positive rate for identifying positive target sites within this restrictive set. However, this would be at the cost of missing out on a large number of positive target sites that are not present in the candidate set in the first place.

For our method, we select the least restrictive filter to have the most expansive superset for the initial selection of possible target locations genome-wide. Specifically, in our case we have the thermodynamic cut-off of –15 kcal/mol, and then, to generate seed-sites, we constrain the seed match to be at least a 6-mer without using any additional constraints. The cut-off value of –15 kcal/mol was the least restrictive among previous work that consider thermodynamic binding [27, 47]. If a target region meets *either* of these two criteria, then we include it in the candidate set. Thus, we challenge our model by coming up with the most expansive set of potential target sites. This shows up quantitatively in Table 1, where we see that the dataset that we evaluate on is the largest among all prior work.

**Table 1:** Comparison of the normalized candidate set size used by various methods

	mirSVR	PITA	TargetScan	STarMir	*Avishkar*
Human	1.256	3.078	0.181	56.183	66.081
Mouse	0.56	2.179	0.318	37.418	75.503

The normalized candidate set size is obtained by dividing the candidate set size by the number of miRNA times the number of mRNA. The normalized candidate set size can be interpreted as the average number of candidate sites considered by the method for a miRNA-mRNA pair. mirSVR, PITA, and TargetScan only consider the 3’ UTR region, so the normalized candidate set size is very low for those methods

Algorithm 1 describes our process of generating the candidate set. Line 3 of the algorithm extracts all the candidate target regions for an miRNA-mRNA pair for which the binding energy (*ΔG*) is less than –15 kcal/mol. Line 4 of the algorithm extracts all target sites which have at least a 6-mer seed match. Line 5 removes from the entire set of target sites (extracted in step 3), those target sites that also have a seed match. Finally, the algorithm returns a set of seed and seedless target sites for each miRNA-mRNA pair.



Since different methods use different numbers of miRNAs and mRNAs, we use a metric called *“normalized candidate set size”* to compare against other methods. The normalized candidate set size is defined to be the size of the candidate set divided by the product of number of miRNAs and mRNAs used by the method. So, the normalized candidate set size can be thought of as the average number of potential target sites considered by a method for a miRNA-mRNA pair. *The higher the number, the more general and less restrictive the method is.*

**Fig. 1:**
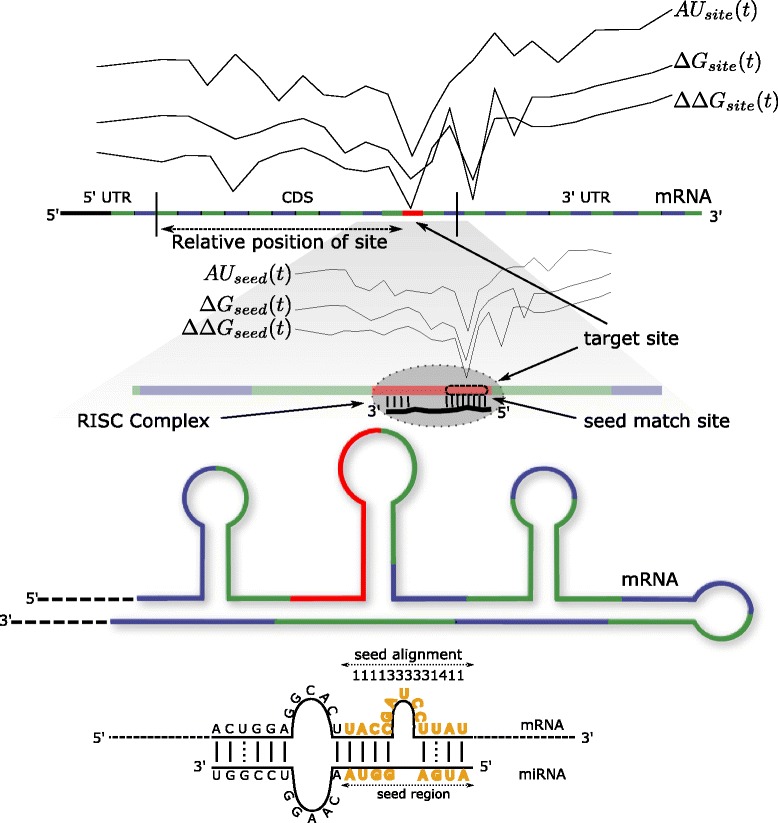
A schematic of some of the features used in the model. The alternating blue and green regions denote the 13 consecutive windows around the target site (shown in red), where various thermodynamic and sequence features are computed. The seed alignment pattern is computed by considering the mRNA nucleotides that are aligned with nucleotides from index 1 to 8 from the 5’ end of the miRNA. The alignment is represented by a vector of 1 (match), 2 (mismatch), 3 (gap), and 4 (GU wobble). The relative position of the target site within an mRNA region and the length of the target site are also used as features

After generating the candidate set, we use CLIP-seq datasets to label the positive (that is, functional) sites. Specifically, if the site is contained within an AGO-crosslinked region for the mRNA, we label the mRNA fragment to be a (positive) target site. Thus, *miRNA-mRNA interactions are deemed positive (functional) if the target site is present in an AGO-crosslinked region and, additionally, either the binding energy of the miRNA-mRNA hybrid is below a certain threshold or if there is a seed match.* In this paper, we use the term *“seed match”* to refer to a perfect pairing within nucleotides 1 to 8, of length at least 6, from the 5’ end of the miRNA. We call the corresponding mRNA target site as a *“seed site”*. On the other hand, any pairing within nucleotides 1 to 8 from the 5’ end of the miRNA that does not involve at least a perfect 6-mer match is referred to as a *“non-canonical seed match”*; the corresponding mRNA target site called a *“seedless site”*. ^5^ While our method makes no distinction between a canonical and non-canonical seed match, we do present results for seed and seedless sites separately. This is in order to gain more insight into the subtleties of miRNA target recruitment, as demonstrated in Fig. 1 (there could be perfect matches, mismatches, gaps, or GU wobbles, for example), as well as to compare the performance of our algorithm against other methods that generate predictions exclusively for sites involving canonical seed matches. Notably, in the actual execution of our method, we use a principled approach for all target sites, whether seed or seedless, by calling into play a novel metric that we call *seed enrichment metric*, which we describe later in the Methods Section.

Thus, given the notion of a functional miRNA-mRNA interaction, the learning problem becomes that of predicting whether a given region in an mRNA is targeted by a miRNA.

### Results

**Fig. 2:**
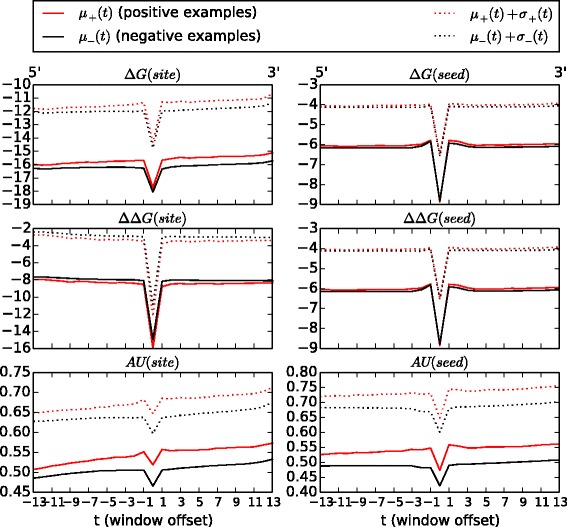
The mean curves (*μ*(*t*)), plus 1 × standard deviation (*σ*(*t*)), for various curves in the positive (red) and negative (black) miRNA-mRNA set for the human dataset (PAR-CLIP). The mean and standard deviations were computed for each index −13≤*t*≤13 over all examples. A window offset of 0 corresponds to the target site in the mRNA while other offsets correspond to positions of the moving window on either side of the target site. The mean is computed over examples where the entire curve was available, i.e., discarding the cases where the matching region was toward one end of the mRNA

By representing the features *ΔG*, *ΔΔG*, and local AU content as curves (Fig. 2), some interesting patterns immediately emerge. For instance, we see that all three features, on an average, have a characteristic V-shape, where the value of the feature has a steep dip at the target site. This is partly due to fact that the target site mostly has a smaller size than the size of the consecutive windows that we use to compute values at regions flanking the target site. For all the features, we do notice that there is significant overlap between the values of curves for positive and negative miRNA-mRNA interactions—observe the overlap in Fig. 2 between the mean + standard deviation of the positive and the negative samples. What is interesting though is that, among the three features, the curves for local AU content have the most separability for the positive and negative examples.

Another subtle difference between positive and negative miRNA-mRNA interactions is that for AU content and *ΔG*, the value of the curves gradually increases from the 5’ to the 3’ end. Also, the rate of increase is greater for the positive examples than for the negative examples.

Finally, we note that the difference in binding energy (*ΔG*) is sharply lower at the target site compared to that at the flanking regions. The difference becomes increasingly pronounced as we move from the 5’ end to the 3’ end of the mRNA. So the curves for *ΔG* seem to suggest that the one of the factors that determines if a miRNA will target a certain mRNA region is governed not so much by the stability of the miRNA-mRNA duplex at the target region but more by the difference in duplex stability between the target site and the target-flanking regions. A larger difference between duplex stability at the site and flanking regions translate to a greater preference for binding. Thus, given the differences between positive and negative miRNA-mRNA interactions, in terms of the various thermodynamic and sequence (AU) curves, it only seemed natural to incorporate some sort of representation of these curves into our model. Toward that end, we use non-parametric representations of the curves. These curves are represented as linear combinations of cubic B-spline basis functions with only very general smoothness assumptions. Specifically, for a B-spline, the assumption is that the second derivative of the curve exists and is continuous everywhere. In the next section, we describe the performance of our methods *vis-à-vis* competition.

### Comparison against other methods

For comparison with competition, we use the CLIP-seq datasets, which have mRNA information, and coarser-grained (larger) nucleotide regions that include the actual AGO binding site, call that: *l*_1_. For our synthetic data, which is generated from the experimental data, we have: (mRNA, miRNA, *l*_2_), where the location *l*_2_ is finer-grained and is localized within *l*_1_. We give competitive protocols a victory, if they predict a binding site as: (mRNA, miRNA, *l*_3_), if *l*_3_ has at least a threshold amount of overlap with location *l*_1_ (AGO-crosslinked region). The threshold that we use for our evaluation is 90 % ^6^. Note that this gives competition the benefit. Further, let us consider the following scenario: if the actual synthetically-generated data is (mRNA-a, miRNA-b, *l*_2_) and the prediction from competition is (mRNA-a, miRNA-c, *l*_2_), we count that up as a victory for the competitive protocol. So, our evaluation procedure does not penalize other methods if the identity of the miRNA is different for a target site than what we have computed.

**Fig. 3:**
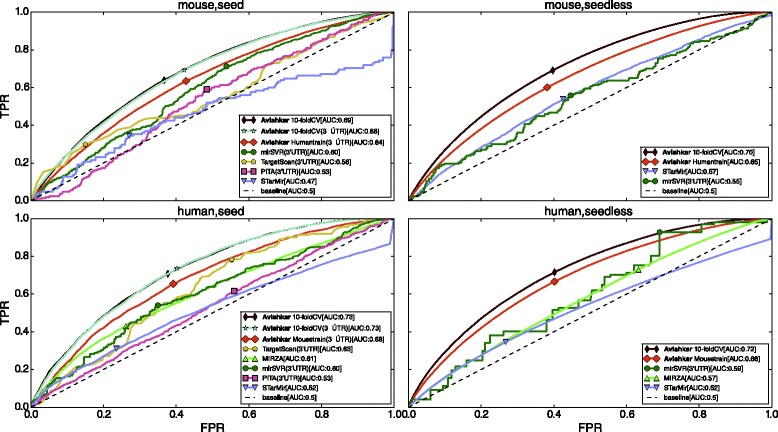
ROC curves for Human (PAR-CLIP) and Mouse (HITS-CLIP). The figures in the first row are for target sites involving canonical seed matches while the second row shows results for non-canonical seed match target sites. The legend "Human train" in the ROC curves for mouse data indicates the model which was trained on human data while the mouse data was used as the test dataset. Similarly the legend "Mouse train" in the ROC curves for human data indicates the model which was trained on mouse data while the human data was used as the test dataset. MirSVR [26], PITA [36], and TargetScan [37] only generate predictions for seed match sites in the 3’ UTR region. Note that for seedless sites in human, although mirSVR appears to perform slightly better than MIRZA, it generates very few seedless target sites, thereby resulting in a very jagged ROC curve. The markers indicate points on the curve where the difference between the TPR and FPR is maximum

Figure 3 shows the 10-fold cross-validation as well as cross-species prediction performance of our algorithm *vis-à-vis* mirSVR [26], PITA [32], TargetScan [27, 33], and MIRZA [28], on the human and mouse datasets. Since mirSVR, TargetScan, and PITA, only consider the 3’ UTR region for making predictions, to have a fair comparison, we also validated our model only for those target sites that are present in the 3’ UTR region.

From Fig. 3, it is clear that our method outperforms all competition for all the genic regions. We note that the curves for *Avishkar* are smoother because they have been averaged over multiple hold-out datasets during cross-validation and sub-sampling from the larger negative dataset. Further, another factor contributing to the smoothness of our ROC curves is the fact that our model has very low variance at the cost of increased bias. This is further discussed in the Discussion Sub-Section.

There are only a few methods that predict non-canonical target sites. We are able to get better performance for these non-canonical site predictions as well. Also, the difference in performance between intra-species and inter-species prediction is quite small, indicating that our method performs quite well in predicting across species, and by extrapolation, across multiple cell types.

### Performance evaluation on experimentally validated mRNA-miRNA interactions

We also evaluated the quality of our predictions against experimentally validated positive miRNA-mRNA interactions obtained from the miRTarBase database [53]. Since the miRTarBase database has very few experimentally validated non-functional miRNA target interactions (MTIs), we compared the predictions against functional MTIs only and are thus able to calculate the recall metric value. For mouse, using a threshold of 0.5 on the probability scores output by *Avishkar*, (greater than the threshold means we conclude the interaction is functional) we were able to successfully predict 1,942 functional MTIs out of 2,445 functional MTIs available in the miRTarBase database for the mRNAs and miRNAs evaluated in our method. This amounts to a recall value of 79.4 %. Similarly for humans, our method was able to predict 895 out of 914 functional MTIs, amounting to a recall value of 97.9 %.

### Performance on unseen mRNAs

From the features that we generated for 1,200 mRNAs for humans and 4,000 mRNAs for mouse, we wanted to check how our method would perform on mRNAs that it had not seen before. To answer that question, we trained our method on features generated for a subset of mRNAs and evaluated the performance of the model on the remaining mRNAs. We started out by training on only 1 % of the mRNAs and progressively increased the fraction of mRNAs used in training. Figure 8 shows the average ROC curves obtained for human mRNAs. We see that when training on only 1 % of the mRNAs, our model achieves performance close to the full set of mRNAs—AUC is only 7 % less. The performance of our model quickly saturates (at around training size of 20 %) and adding data for more mRNAs in the training set does not increase the predictive power of our model. This is evidenced by the fact that the curves for training sizes of 20 %, 40 %, and 60 % are all overlapping. This further alludes to the fact that the performance of our model is limited by bias and not due to over-fitting on the training data. From this experiment, we conclude that our method generalizes well to unseen examples.

### Performance on miRNAs that are not abundantly expressed

There are two factors that determine if a miRNA will target a mRNA segment and hence show up in a CLIPed region. One being the affinity of the miRNA for the mRNA segment and the other is the relative abundance of the miRNA in the cell-line (the prior probability). In our model we try to learn the affinity of a miRNA for a mRNA segment from CLIP-seq data and to do that it is important to eliminate the other factor, i.e. the prior probability of a miRNA targeting a mRNA fragement due to its relative abundance (or lack thereof) in the cell-line. That is why it is important to train the model on the most-frequently expressed miRNA families. However, once the model is trained it can be used to predict for miRNAs that are not adequately expressed. To validate that hypothesis we generated target site predictions for the human miRNA family miR-99, consisting of four different miRNAs, for all 9158 human mRNAs by training on the 10 most-abundantly expressed human miRNAs. We validated the predictions against data from miRTarBase database. We were able to successfully predict 134 out of 155 MTIs in the miRTarBase database for the miR-99 miRNA family, thereby achieving a recall value of 86.5 %. This shows that our model can be used to generate accurate predictions for miRNAs on which the model wasn’t trained.

### Importance of various features

Figure 5 shows the feature weights learned by our model for both canonical seed sites and seedless sites. Negative weights for a scalar feature correspond to it being negatively correlated with the positive miRNA-mRNA interactions. In contrast, for functional covariates, it is difficult to interpret the “sign” of the weights for the B-spline basis functions. This is because the coefficients of the basis functions control the shape of the curve, where, larger *absolute* weights correspond to higher predictive power. Table 3 ranks the top 20 features in descending order of the absolute value of their weights. For functional covariates, the number within square braces indicates the coefficient index in the B-spline basis function expansion of that feature.

**Fig. 4:**
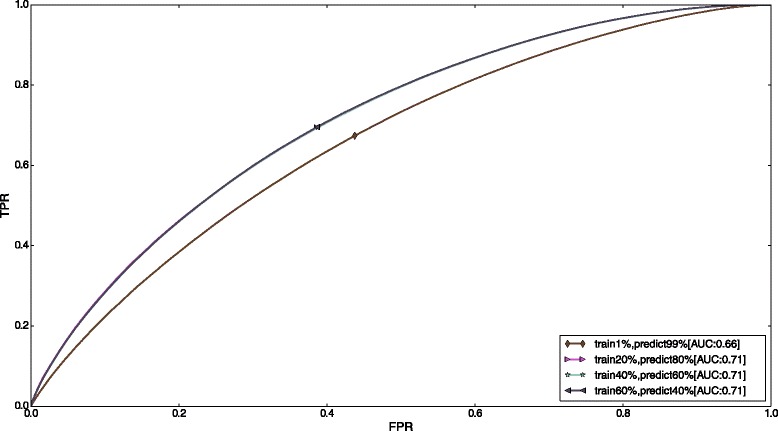
ROC curves for 10-fold cross-validation performance of *Avishkar* in different regions of the gene in the human dataset

**Fig. 5:**
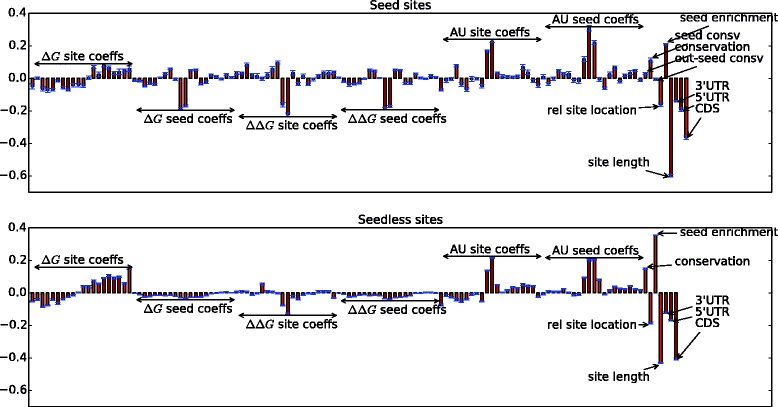
Mean feature weights (along with standard deviation) learned by the linear SVM model for seed and seedless sites

**Table 2:** Attributes of data used for training and prediction in Avishkar

	# Positive examples (Seed:Seedless)	# Negative examples	# mRNA	# miRNA	# Positive target sites in		
					3’ UTR	CDS	5’ UTR
HITS-CLIP (Mouse)	861,208 (6 %:94 %)	35,608,333	4,059	119	478,138 (≈56 *%*)	367,371 (≈43 *%*)	15,699 (≈1 *%*)
PAR-CLIP (Human)	141,109 (8 %:92 %)	2,659,748	1,211	35	80,775 (≈57 *%*)	55,250(≈39 *%*)	5,084 (≈4 *%*)

For both mouse and human data, most of the positive miRNA target sites are found in the 3’ UTR region, followed by the CDS region, with very few target sites located in the 5’ UTR region

**Table 3:** Relative importance of features for seedless and seed sites

	Seedless Sites		Seed Sites	
Rank	Feature	Weight	Feature	Weight
1	CDS	–0.452	Site length	–0.591
2	Site length	–0.404	CDS	–0.436
3	Seed enrichment	0.364	*A* *U*_*seed*_ [9]	0.287
4	*A* *U*_*site*_ [10]	0.198	*Δ* *Δ* *G*_*site*_ [10]	–0.250
5	*A* *U*_*seed*_ [10]	0.198	*A* *U*_*seed*_ [10]	0.235
6	*A* *U*_*seed*_ [9]	0.192	Seed enrichment	0.210
7	5’ UTR	–0.165	*A* *U*_*site*_ [9]	0.208
8	Consv	0.149	*Δ* *G*_*seed*_ [9]	–0.195
9	*Δ* *Δ* *G*_*site*_ [10]	–0.148	*Δ* *Δ* *G*_*seed*_ [9]	–0.193
10	Relative site location	–0.141	*Δ* *Δ* *G*_*seed*_ [10]	–0.190
11	*A* *U*_*site*_ [9]	0.134	*Δ* *Δ* *G*_*site*_ [9]	–0.187
12	*Δ* *G*_*site*_ [15]	0.118	*A* *U*_*site*_ [10]	0.187
13	*Δ* *G*_*site*_ [19]	0.116	*Δ* *G*_*seed*_ [10]	–0.179
14	*Δ* *G*_*site*_ [14]	0.113	5’ UTR	–0.178
15	*Δ* *G*_*site*_ [17]	0.111	Seed consv	0.175
16	*Δ* *G*_*site*_ [16]	0.098	Relative site location	–0.154
17	*Δ* *Δ* *G*_*site*_ [9]	–0.093	*Δ* *G*_*site*_ [12]	0.100
18	*A* *U*_*seed*_ [8]	0.089	*Δ* *G*_*site*_ [14]	0.097
19	*A* *U*_*seed*_ [11]	0.088	3’ UTR	–0.089
20	*Δ* *G*_*site*_ [12]	0.078	*A* *U*_*seed*_ [3]	0.088

The rank is computed by sorting by absolute value of weight in descending order. For functional covariates, the numbers in square braces indicate the coefficient index for the B-spline basis functions

It is immediately evident from Table 3 that for seed sites, most of the B-spline basis function coefficients, with the exception of AU content, correspond to the “seed” curves. While, for the seedless sites, the “site” curves are more effective in differentiating positive mRNA-miRNA interactions from negative ones. This goes on to show that when there is a seed match, the thermodynamic profile of the mRNA seed region is what matters more in determining functional binding sites.

Local AU content is a strong differentiator of positive miRNA-mRNA interactions from negative ones. The weights learned by our model corroborate the conclusion from Fig. 2—since AU curves for positive and negative miRNA-mRNA interactions have the least amount of overlap compared to other features like *ΔG*, *ΔΔG*, etc., they are strong indicators of miRNA-mediated downregulation. In fact, local AU content curves are among the top 5 features for both seed and seedless sites. The fact that the local AU content is one the most important predictors for miRNA target prediction has also been confirmed by mirSVR [26] (Supplementary Figure S1) and, to some extent, by the Random Forest Model, described in [47]. Notably, our representation of local AU content is able to extract significant signal from the feature, which is otherwise missed by scalar representations of the feature. Further, we are also able to capture other spatial characteristics of the feature e.g., the slope of the curve.

Another interesting observation is that for seed sites, accessibility (*ΔΔG*) of the target site is a better indicator of miRNA-mediated downregulation of mRNA than the thermodynamic stability of the miRNA target duplex (*ΔG*). This is evident from the number of *ΔΔG* coefficients showing up in the top 20 features for seed sites. On the other hand, for seedless sites, *ΔG* coefficients dominate in the top 20. Kertesz *et al.* [32] argue that accessibility along with binding energy is a better indicator of miRNA targeting than binding energy alone (*ΔG*). However, from our results this appears to be the case more often for seed sites. One possible explanation for this might be that the binding free energy (*ΔG*) is mostly similar for different classes of seed matches (6-mers, 7-mers, and 8-mers). So, for seed sites, accessibility of the target region (*ΔΔG*) becomes the major discriminator between positive and negative miRNA-mRNA interactions. In contrast, for (non-canonical) seedless sites, where there may be little base pairing at the seed region, the binding free energy becomes the limiting factor.

**Fig. 6:**
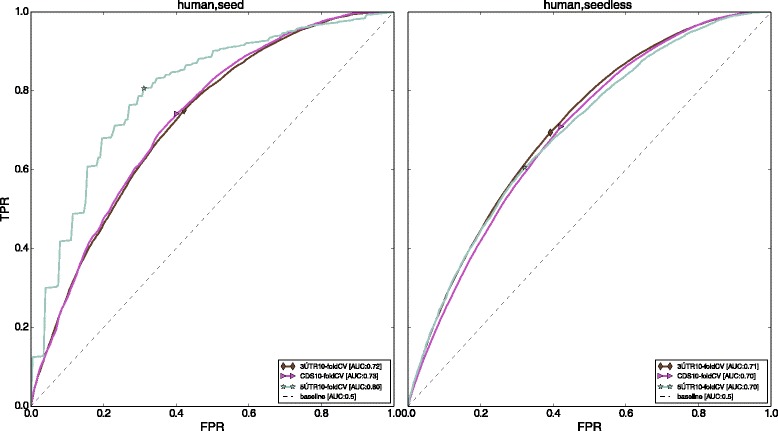
Scatter plot of frequencies of various types of seed alignment patterns in set of positive miRNA-mRNA interactions for Mouse (x-axis) and Human (y-axis). Among the top-10 most frequently occurring patterns, only two, namely, the 6-mer and 7-mer, are canonical, seed-match patterns. In the labels for the top-10 most frequently occurring patterns, 1 indicates a match, 2 indicates a mismatch, 3 a gap, and 4 indicates a GU wobble

We notice that our metric, seed enrichment, is also an important indicator of miRNA targeting. In fact, seed enrichment is among the top three features for seedless sites. We are able to get more signal from the seed enrichment feature for non-canonical sites than for canonical sites. This is because there are only a few different types of seed-match patterns for canonical seed sites, all with high values of enrichment. On the other hand, for non-canonical sites, seed enrichment varies greatly between different types of non-canonical interaction patterns. Figure 6 shows the proportion of various types of seed-match patterns, canonical and non-canonical, in the positive dataset for human and mouse. We see that the occurrence frequency of various patterns in the human and mouse data is highly correlated, as indicated by the correlation coefficient of 0.923. This indicates that the various patterns of canonical and non-canonical seed matches are conserved across species, rather than occurring merely by chance. What is also surprising is that among the top-10 most frequently occurring patterns, only two are canonical seed matches, namely, a 6-mer and a 7-mer match. Other frequently occurring seed-match patterns have long bulges, as indicated by a series of gaps (denoted by 3s).

We also note that the prediction performance for 3’ UTR sites is almost identical to those of other sites (see Fig. 4)^7^. This goes on to show that the inclusion of the categorical feature indicating the type of region, namely, 3’ UTR, CDS, or 5’ UTR, is able to explain the differing efficacies of target sites in different regions, and that other features like thermodynamic binding, accessibility, conservation, etc., have the same predictive power in the three different regions.

It should be noted that Table 2 indicates that a large number of positive target sites, around 40 % for both human and mouse datasets, are present in the CDS region. However, our model aggressively tries to label those target sites as negative sites—note the large negative weight for the CDS regions in Fig. 5. This hints at one of two possibilities. First, a lot of the target sites reported by the CLIP-seq methods in the CDS region may be due to transient protein-binding events and, thus, the level of downregulation due to such binding sites may not be significant [18, 54, 55]. Alternately, the mechanism of miRNA action in the CDS region is different from that in other regions and that other features (or methods) might be needed to explain miRNA targeting in the CDS region.

Finally, we draw attention to the weights learned for conservation, site length, and the relative position of a site within one of the 3 regions, namely, 3’ UTR, 5’ UTR, and CDS Table 3. It is evident that conservation plays a positive, albeit small, role in determining true miRNA binding sites. Again, for canonical seed sites, conservation of the seed region (seed consv) is more important than conservation of the overall mRNA target site (consv). We note that since we use conservation as a feature (one among many used in our SVM classifier), as opposed to using it as a filter, like some methods have done in the past [56, 57], we are also able to predict target sites that are *not* conserved. The length of the target site is also strongly anti-correlated with the probability of a site being a true binding site which shows that shorter miRNA-mRNA alignments, i.e., miRNA-mRNA alignments with fewer gaps or bulges, are preferred.

### Difference between 3’ UTR and 5’ UTR binding patterns for seedless sites

**Fig. 7:**
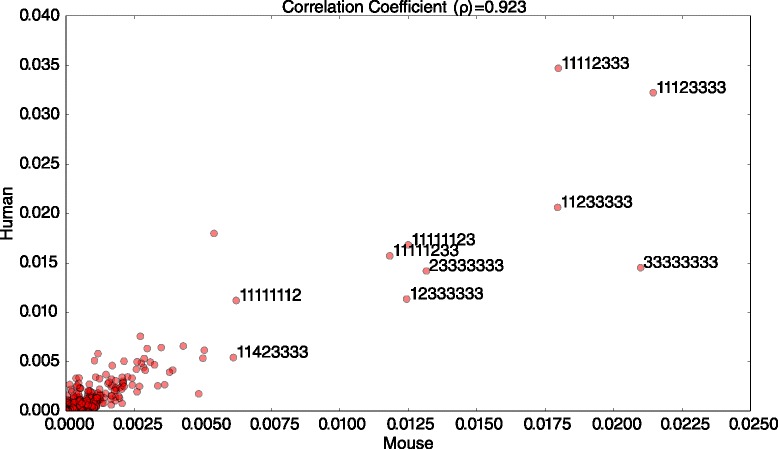
Optimal number of basis functions (*K*) computed using 10-fold cross-validation and Gaussian process regression

In order to understand the difference between 3’ UTR and 5’ UTR binding patterns for seedless sites, we analyzed the weights learned by our model for the two regions^8^. The results are given in Fig. 9. There are two important differences between 3’ UTR and 5’ UTR binding patterns. One, conservation plays a much more important role in the 3’ UTR region in determining the positive target sites, compared to the 5’ UTR binding region. This is evident by the large positive weight for conservation in the 3’ UTR. This result is widely known in the literature [58]. Second, where the matching location is found differs between the 3’ UTR seedless site and the 5’ UTR seedless site. For the 3’ UTR, the matching location is more likely to be toward the beginning while for the 5’ UTR, it is more likely to be toward the end. The evidence for this comes from Fig. 7 for the “relative site location” feature which takes a value between 0 and 1, with 1 indicating the 3’ end.

**Fig. 8:**
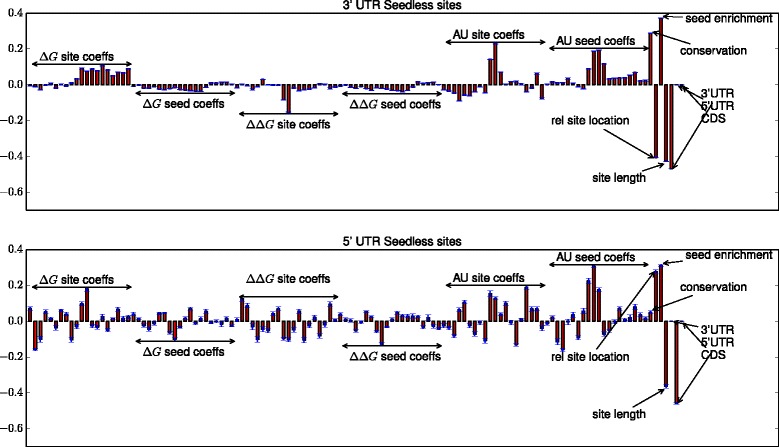
Performance of model on V-CLIP data by progressively varying the fraction of mRNAs used for training and prediction. In the first case, data for 1 % of the total mRNAs (around 1200) was used for training and data for the rest of the mRNAs was used to evaluate the model. The percentage of mRNAs used for training was then progressively increased to 20 %, 40 %, and finally 60 %. These three curves are overlapping, indicating that there is no performance improvement by increasing the training size beyond 20 %

### Discussion

The number of basis functions (*K*), controlling the smoothness of the various curves, used in our model is the only other tunable parameter, apart from the regularization parameter (*λ*). We set a very low value for *λ* because of our use of a simple linear model that avoids overfitting anyway. We choose the value of *K* using 10-fold cross-validation to maximize the difference between TPR and FPR (see Fig. 7). So, in effect, by fitting a smooth curve through noisy observations, and using the coefficients of the basis functions as features, we reduce the dimensionality of the feature space used in our method. Currently, we use a single number *K* for all the curves, which is a simplification done to reduce the parameter space that needs to be searched during training.

We use a linear SVM model, a relatively simple ML classifier. This has the advantage that the model is interpretable, directly from the weights of each feature, and the computational burden is low. However, it displays a bias in its prediction. For example, the misclassification rate of our model remained close to 30 % both during training and during intraspecies and inter-species validation. We are looking to improve on this by using a slightly more expressive model, such as, non-linear SVM model.

Finally, we found that the TPR-FPR performance of STarMir [27] is much worse than that reported in their paper. We contacted the authors multiple times regarding the issue but were unsuccessful in eliciting a response.

### Threats to validity of our approach

In our approach, we have made the assumption that functional miRNA target sites are those that are present within the AGO-crosslinked regions, as identified by CLIP-seq. In that respect, our method has the most agreement with CLIP-seq data, among other computational approaches. However, since the identity of the miRNA present in the CLIP region is unknown, it may happen that the miRNA predicted by our model is different from the miRNA that was actually involved in the binding event. Also, given AGO-crosslinked regions for various mRNAs, we attempt to extract finer-grained target sites within the AGO-crosslinked regions that may be targeted by a set of miRNAs. Toward that end, we only consider the most abundantly expressed miRNAs in a cell-line—top-10 miRNA families for human and top-20 families for mouse data. This choice is as per the prior work [49] for mouse and [50] for human datasets, which state that those families accounted for most (95 % for human) of the miRNA sequence reads. Thus, our method misses out on functional miRNA target sites that may be outside AGO-crosslinked regions or fails to identify mRNA sites that are targeted by the miRNAs whose expression levels are low.

We take the CLIP data as ground truth and that is not completely correct because the CLIP data itself has false positive examples. In future work, we will augment this with other data types, such as, RNA-seq data for gene expression to reduce such false positives.

## Conclusion

In this paper, we have presented an efficient SVM-based model called *Avishkar* for miRNA target prediction utilizing CLIP-seq datasets. *Avishkar* has in its dataset the largest number of potential miRNA-mRNA interaction sets and demonstrates the best performance among other established computational methods for miRNA target prediction. In developing our model, we leveraged the fact that functional miRNA-mRNA interactions have specific spatial thermodynamic and sequence profiles. We used non-parametric representations of curves, in the form of cubic B-spline basis functions, in order to represent these contributory features, such as thermodynamic and sequence features. This is in contrast to traditional methods that rely on simplistic scalar representations of the features. We further unified canonical and non-canonical seed matches into a single model and were able to demonstrate that a lot of non-canonical seed match patterns are, in fact, enriched in the set of functional miRNA-mRNA interactions. Our Area-Under-the-Curve for the ROC curve for both human and mouse datasets are better than all prior work, quantitatively 19.7 % and 22.0 % better for human (seed and seedless respectively) and 15.0 % and 22.8 % for mouse (seed and seedless respectively). We conclude by noting that further experimental or computational analysis of the functional sites predicted by our algorithm is needed to confirm the identity of miRNAs involved in the protien (AGO) binding events and to quantify the amount of repression of different genes by those miRNAs.

## Methods

### Data

The characteristics of the data that we used are summarized in Table 2.

We downloaded CLIP-seq data for the human HEK 293 cell line, a human embryonic kidney cell line, published by [50] from Gene Expression Omnibus (series GSE28865). To be specific, we used the three datasets having codes GSM714642, GSM714644, and GSM714646, which correspond to samples from replicate A experiments involving AGO2 protein. The datasets identify 40 nucleotide-long AGO binding sites for each mRNA. The combined data from all three datasets contained 190,764 AGO binding sites across 10,159 different mRNAs. Following the same approach as [50], we used the 10 most abundantly expressed miRNA families, comprising of 44 different miRNAs, in human HEK 293 cells, to identify miRNA-mRNA binding sites. Since the total number of potential binding sites of 44 miRNAs across 10,159 mRNAs is enormous and then generating features for all those binding sites is computationally expensive, we randomly selected around 1,200 different mRNAs to train and evaluate our model.

For mouse, we downloaded HITS-CLIP data obtained from the mouse brain tissue [49], from the starBase database [59, 60]. The data contained locations of 11,117 AGO-CLIP tags (actually CLIP tag clusters) in the mouse genome (mm9 assembly) in a BED file. We mapped chromosome coordinates to mRNA locations by first extracting the nucleotide sequence for the corresponding chromosome segment from the UCSC DAS server using the provided REST API.

Then we use NCBI BLAST to map those sequences to mRNA names (and locations) using the RefSeq RNA database. As in [49], we used the 20 most abundant miRNA families, containing 119 miRNAs to identify miRNA-mRNA binding sites. We obtained the miRNA names and sequences from the Supplementary Data provided by the authors [49]. We then generated the candidate set of miRNA-mRNA interactions using Algorithm 1. After that, we labeled each target location in the candidate set as 1 or 0, depending on whether the candidate location for an mRNA was contained within an AGO-crosslinked region or not.

To incorporate evolutionary conservation of genome regions of human and mouse genomes, we downloaded PhastCons [61, 62] conservation scores from the UCSC Genome browser. For mouse, we used the conservation scores generated by alignment of 30 vertebrate genomes to the mouse genome (mm9 assembly). Similarly for human, we used the conservation scores generated by alignment of 44 vertebrate genomes to the human genome (hg18 assembly).

To compute the extents of various mRNA regions like 3’ UTR, 5’ UTR, and CDS, we downloaded annotations for hg18 and mm9 assemblies from the UCSC Table browser (RefSeq Genes track). Mature miRNA sequences were downloaded from the miRBase [63] website [64]. In subsequent sections, we describe the various features considered in our model.

During cross-validation, we use all the positive samples and sub-sample to create an equal number of negative samples. During all intra-species experiments, we use 9/10-ths of the data for training and the rest 1/10-th for prediction. During inter-species runs, we use the entire dataset’s positive examples and an equal number of negative samples, from species 1 to predict the entire dataset for species 2.

### Thermodynamic features

Thermodynamic stability of the miRNA-mRNA target duplex have long been identified as being an important predictor of true binding sites of a miRNA [4, 32]. Thermodynamic stability of the miRNA-mRNA duplex is given by the free energy gained by binding of miRNA to the target site and is denoted by *ΔG*. Thermodynamic accessibility has also been argued to be an important predictor of miRNA repression [32]. Accessibility is defined as the “difference between the free energy gained by the binding of the miRNA to the mRNA (*ΔG*) and the free energy lost by unpairing the target site nucleotides, *ΔG*_*open*_” [32]. The target site nucleotides need to be unpaired to make the site accessible to the RISC complex housing the miRNA, so *ΔΔG* measures the effective accessibility of a region. We consider both the features in our model; however previous work that consider these features [26, 47], compute the *ΔG* and *ΔΔG* values either at the target site or at the target site along with upstream and downstream flanking regions of a given length. Following this, they use the features as scalar covariates into a classification or regression model. Liu *et al.* [27] increase the length of the flanking region in discrete chunks of five nucleotides. However, all such prior characterizations of target site accessibility oversimplify the spatial nature of miRNA-mRNA interaction. For example, as shown in the illustration in Fig. 1, the target site might be surrounded by tight secondary structures, which make it difficult for the miRNA-RISC complex to interact with the target site. So, we had the idea that characterizing the thermodynamic interactions as curves, and taking into account the shape of the curves in our model, would improve the model’s predictive power. Toward this end, we take a different approach to characterizing the thermodynamic stability of miRNA-mRNA duplex and the accessibility of the target site. We consider the thermodynamic profile of miRNA-mRNA interaction by taking into account *ΔG* and *ΔΔG* values at the target site and use 13 consecutive windows, both upstream and downstream of the site region, of size 46 nucleotides each. However, rather than treating them as separate features to be fed into a classifier, which effectively discards the spatial nature of the phenomenon, we fit smooth curves through the noisy observations to define what we call the **“thermodynamic curves”**. The smoothed thermodynamic curves are used as functional covariates in our model. Here, the word functional is used in the statistical sense, meaning that the features are infinite-dimensional functions, as opposed to being finite-dimensional vectors. The window length was chosen to be 46 nucleotides because the AGO footprint on the mRNA spans around 46 nucleotides [49]. The various curves used in our model that are centered at the target site and computed at a resolution of 46 nucleotides are collectively referred to as “site curves” in our paper. Further, since the dimensionality of the curve is not known *a priori*, we use basis functions to achieve a “good-enough” fitting curve. We experiment with different numbers of basis functions and settle on the optimal number, 20, through our training phase. Our results show that computing thermodynamic profiles of miRNA-mRNA interactions in terms of these curves, as in Fig. 2, captures richer information than computing binding and accessibility energy at the target site alone. We are able to discriminate the signatures of the binding sites better due to the use of curves. Further, we are able to extract the relative importance of the curves for the various thermodynamic features, as also for the local AU content feature, through our feature-analysis phase.

We also compute the thermodynamic curves at a finer resolution, collectively referred to as “seed curves” in the paper, by using a window of size 9. We compute the binding (*ΔG*) and accessibility (*ΔΔG*) curves centered at the mRNA seed-matched region, i.e., nucleotides of the mRNA that are paired with the seed region of the miRNA, along with 13 consecutive windows both upstream and downstream of the seed-matched region. The rationale underlying this is that pairing of nucleotides 1–8 from the 5’ end of the miRNA has been deemed to be much more functional than pairing at other nucleotide regions. For target sites, where there is no seed match (i.e., a 6, 7, or 8-mer), we pick the region within the target site that has the most favorable hybridization with nucleotides 1–8 of the miRNA as the seed-matched region. Thus, the seed curves capture the thermodynamic signatures for the mRNA seed region. It should be noted that the thermodynamic curves considered in this paper are fundamentally different from those computed by [27], where they compute thermodynamic values by keeping the window centered at the target site region. They then increase the window length on either site of the target site region in increments of 5 nucleotides. Further, the different values computed for *ΔG* and *ΔΔG* are not factored in as curves. Rather, they are fed as separate scalar features into a classifier. The authors do not provide any interpretation of the nature of information that such features capture nor are they able to demonstrate the usefulness of these features. The only relevant features, as reported by the authors in their website [65], appear to be thermodynamic features computed at the target site alone.

### Seed match enrichment

Bartel *et al.* [4] defined a hierarchy of five different types of miRNA seeds that roughly correspond to the miRNA’s efficacy in downregulating mRNA targets. So, a lot of computational approaches for miRNA target prediction use the seed type as a categorical feature in their model.

In fact, Xu *et al.* [47] state that due to the difficulty of incorporating various patterns of insertions and deletions that may occur in the seed-matched region, they only consider one type of non-canonical seed match by allowing a single GU wobble. Indeed, a model that enumerates all possible patterns of seed matches, and tries to learn the importance of each type of pattern in mRNA downregulation, would perform poorly because of the sheer number of possible patterns. We circumvent this problem by representing the alignment of a miRNA with an mRNA as a vector, where each element takes four possible values corresponding to a match, mismatch, gap, and GU wobble respectively. We come up with a metric called **“seed enrichment”** that captures, in a single numeric feature, the relative efficacy of various kinds of seed matches. We observed that a vast number of seed matches, having long bulges (gaps) were enriched, providing further justification for our consideration of non-canonical seed matches. This observation is also corroborated by [25].

*Enrichment score for each seed match.* We precompute the number of occurrences of various seed-match patterns in the positive miRNA-mRNA interaction dataset and the corresponding seed enrichment score for each pattern as follows. Let us consider the likelihood that a particular pattern of seed match, **a**, is positively correlated with miRNA repression. To do this, we calculate the following probability for a given seed match pattern, **a**, which has say *k* occurrences among *n* positive samples. Let *α* be the probability that there are *k* occurrences of pattern **a** among *n* samples purely by chance. As an example, for a region of length |**a**|, the expected number of pattern matches **a** in *n* samples, purely by chance will be 0.25^|**a**|^*n*. Then, *α* is given as:

(1) $$\begin{array}{*{20}l} \alpha &= Binomial\left(k | n, 0.25^{|\mathbf{a}|}\right).  \end{array} $$

We call 1−*α* our enrichment score.

(2) $$\begin{array}{*{20}l} enrichment(\mathbf{a}) &= 1 - \alpha.  \end{array} $$

The advantage of our method is threefold. First, we are able to consider a lot of different types of seed matches (both canonical and non-canonical) that are enriched in the set of positive miRNA-mRNA interactions in a unified and principled manner. Second, since the overwhelming majority of positive miRNA-mRNA interactions involve non-canonical seed matches, we are able to generate high quality predictions for a lot of target sites that are missed by other methods. Finally, since ML methods typically handle numerical features better than categorical features, especially those with high cardinality, our process of creating a numeric (probability) value allows us to get high accuracy on predictions for non-canonical sites.

### Sequence features

To incorporate sequence features in our model, we consider the functional version of another popular feature: local AU content, which is defined as the fraction of adenine nucleotides (A) and uracil nucleotides (U) in a block of mRNA. Grimson *et al.* [33] showed that the local AU content is weakly correlated with reduced mRNA expression levels. In contrast, by considering AU curves, we are able to extract significant signal from this feature. In fact, as shown in (Table 3), local AU composition is a top feature in our model. Again, like thermodynamic features, we compute the local AU content at two resolutions— site (window length 46) and seed (window length 9).

### Conservation

Evolutionary conservation has been used in the past to reduce the false positive rate of computational miRNA target prediction methods [66]. So, we incorporate conservation scores of the overall target mRNA site, the seed match site, and the off-seed site (nucleotides other than those that are aligned with the seed region of the miRNA)s as additional features into our model. The latter two features are used only for sites containing canonical seed matches, i.e., either a perfect 6-mer, 7-mer, or an 8-mer site.

### Miscellaneous features

Other features used in our model are (a) the region in which the mRNA target site is present, namely, 3’UTR, 5’UTR, and CDS, (b) relative location of the target site within the aforementioned region, on a scale of 0 to 1, where 0 indicates the 5’ end of the region and 1 indicates the 3’ end, and (c) the length of the target site. For long, it had been believed that most of the miRNA targets are located within the 3’ UTR region of the mRNA. However, recently CLIP-seq methods and some other computational methods e.g., [47] have identified functional miRNA targets in other gene regions like the 5’ UTR region and the CDS. So, we use a categorical feature to denote the type of region in which the target site is present and learn weights (importance measures) for the three different genic regions. The relative site location feature might help explain the fact that CLIP tags were enriched near poly(A) sites (i.e., 3’ end) and, to a lesser degree, near stop codons (5’ end), than in the middle of 3’ UTR regions, as reported in [17]. Finally, the feature “site length” accounts for the fact that a perfect pairing between miRNA and mRNA, and hence shorter target site length, might be more preferable than alignments with long bulges on the mRNA (alignment of miRNA nucleotides to gaps), leading to longer target site lengths. The list of features used in our model is summarized in Table 4.

**Table 4:** Summary of features used in our model

*Δ* *G*_*site*_(*t*)	Thermodynamic binding curve centered at the target site obtained by fitting a smooth curve through the vector observation **Δ** **G** ^*site*^.
*Δ* *G*_*seed*_(*t*)	Finer resolution thermodynamic binding curve centered at the seed match region obtained by fitting a smooth curve through the vector observation **Δ** **G** ^*seed*^.
*Δ* *Δ* *G*_*site*_(*t*)	Accessibility curve centered at the target site obtained by fitting a smooth curve through the vector observation **Δ** **Δ** **G** ^*site*^.
*Δ* *Δ* *G*_*site*_(*t*)	Finer resolution accessibility curve centered at the seed match region obtained by fitting a smooth curve through the vector observation **Δ** **Δ** **G** ^*seed*^.
*a* *u*_*site*_(*t*)	Local AU content curve centered at the target site region obtained by fitting a smooth curve through vector observation **a** **u** ^*site*^.
*a* *u*_*seed*_(*t*)	Finer resolution local AU content curve computed at the seed match region obtained by fitting a smooth curve through vector observation **a** **u** ^*seed*^.
Seed enrichment	A scalar feature indicating the extent to which a seed match pattern in enriched in the set of positive miRNA-mRNA interactions set on a scale of 0 to 1.
Site conservation	The extent to which the mRNA site nucleotides are conserved across different species.
Seed conservation	The extent to which the nucleotides in the mRNA site that are paired with the miRNA seed region are conserved across different species. This is only used when there is a canonical seed match.
Off seed conservation	Average conservation score of mRNA nucleotides that are not paired with the seed region of the miRNA. This is only used when there is a canonical seed match.
Target site length	Length of the mRNA target site
Target region	mRNA region where the target site is present, namely, 3’ UTR, CDS or 5’ UTR
Relative position of target site	Relative position of a target site within one of the 3 regions above on a scale of 0 to 1, with 0 indicating the 5’ end and 1 indicating the 3’ end.

The first six are functional covariates (curves) that are obtained by fitting a smooth curve through the vector observations, indicated by bold-faced letters. The rest are scalar covariates. For functional features, the domain of the function is in $\{t: t \in \mathbb {Z}, -13 \leq t \leq 13 \}$

### Feature transformations

In Table 4, the first six features are functional covariates, which are obtained by fitting smooth curves through the vector observations as follows. Let **f**_*i*_ denote one of six feature vectors of length 2*W*+1 for the *i*-th data point, where *W* is the number of windows around either side of the site region. Also, let *f*_*i*_(*t*) denote the corresponding smooth curve. Then,

(3) $$\begin{array}{@{}rcl@{}} f(t) = \sum_{k=1}^{K} c_{i,k} \psi_{k}(t) \end{array} $$

where *ψ*_*k*_(*t*) are the cubic B-spline basis functions. We use the zero value to replace missing values in the vector **f**_*i*_, e.g., when the target site is toward the beginning or the end of the mRNA. The coefficients *c*_*i,k*_ are estimated for each curve by minimizing the least squares error on the discrete observations **f**_*i*_ as follows:

(4) $$\begin{array}{@{}rcl@{}} \mathbf{c}_{i} = \left(\mathbf{\Psi}^{T}\mathbf{\Psi}\right)^{-1}\left(\mathbf{\Psi}^{T}\mathbf{f}_{i}\right) \end{array} $$

where **Ψ** is the (2*W*+1)×*K* matrix of the *K* basis functions evaluated at $\{t: t \in \mathbb {Z}, 0 \leq t < (2W + 1) \}$. This is accomplished by using the interpolation module in SciPy (specifically LSQUnivariateSpline). The number of knots for the cubic B-spline interpolation is computed as *K*−*degree*+2 (degree is 3 for cubic splines). The number of basis functions *K* controls the smoothness of the curve, with smoothness decreasing with increasing *K*. It should be noted that we use the same number of basis functions *K* (≤2*W*+1), and hence, the same smoothness assumptions, for all six functional features, which makes our model slightly restrictive. This is in contrast to a model that uses different numbers of basis functions (and hence different smoothness assumptions) for each of the six functional features. The choice was made to bound the size of the parameter space that has to be explored. Now, for each of the functional features we use the B-spline coefficients as features in the SVM model.

**Fig. 9:**
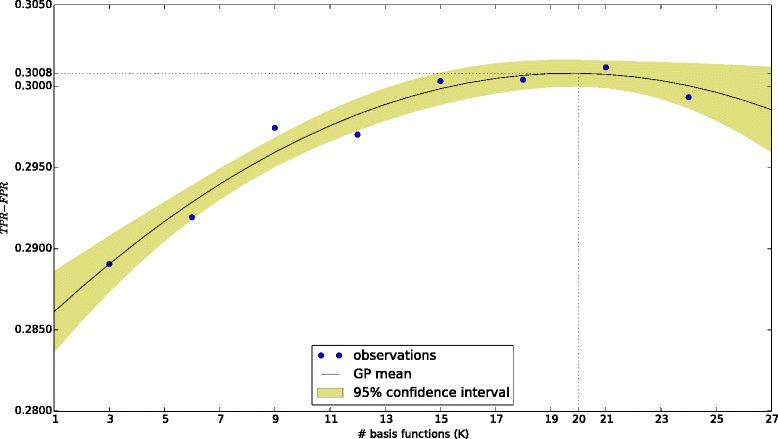
Feature weights for seedless sites in 3’ UTR and 5’ UTR

Let the feature **a**_*i*_ denote the vector that represents the alignment between the first eight nucleotides of the miRNA with an mRNA segment, in the *i*-th data point, as a vector of values, which can be 1 (match), 2 (mismatch), 3 (gap), or 4 (GU wobble), refer to Fig. 9). We precompute the enrichment score for each seed match pattern, as described in Eq. 2. Thus for the *i*-th data point, we lookup the precomputed enrichment score of the seed match pattern **a**_*i*_ and use it as a feature in our SVM classifier.

### Model

Let the training dataset be denoted by $\mathcal {\tilde {D}} = \{y_{i}, \tilde {\mathbf {x}}_{i}\}_{i=1}^{N}$, obtained after transforming the various features, as mentioned in the previous section. We are interested in learning a classifier *f*(*x*_*i*_), such that *y*_*i*_*f*(*x*_*i*_)≥0 (the response variable, *y*_*i*_, for negative examples here is -1 instead of 0, done to simplify notation). We learn a linear classifier i.e., *f*(**x**_*i*_)=**w**^*T*^**x**_*i*_+*b*, by minimizing the loss function given in Eq. 5, using stochastic gradient descent. The loss function in Eq. 5 is the hinge loss and corresponds to a linear SVM. The first term of Eq. 5 penalizes data points that are misclassified (wrong side of the decision boundary) as well as those that are correctly classified points but are too close to the decision boundary, i.e., points within some margin of the decision boundary. Thus, minimizing the loss function results in a maximum-margin decision boundary that best separates the two classes. The second term, called the regularization term, penalizes complex models with large weights.

(5) $$\begin{array}{@{}rcl@{}} L(\mathbf{w}, \mathbf{X}, \mathbf{y}) = \frac{1}{n} \sum_{i=1}^{n} max(0, 1 - y_{i} f(\mathbf{x}_{i})) + \frac{\lambda}{2} ||\mathbf{w}||_{2}^{2}  \end{array} $$

We used Apache Spark [67], running on a Yarn [68] cluster of 10 nodes, to train our model. The regularization parameter *λ*, which controls the trade-off between training misclassification rate and model complexity, is set to a low value of 0.001.

### Validation protocol

We perform the validation of our protocol *Avishkar* on a validation dataset that is distinct from the dataset that was used to train the model. For each example in the test dataset, we compute the probability score, with feature vector **x**^∗^, using the weights learned from training, according to the logistic function given in Eq. 6.

(6) $$\begin{array}{@{}rcl@{}} p(y^{*} = +1) = \frac{1}{1 + \exp\left(-\mathbf{w}^{T}\mathbf{x}^{*} - b\right)}  \end{array} $$

**Fig. 10:**
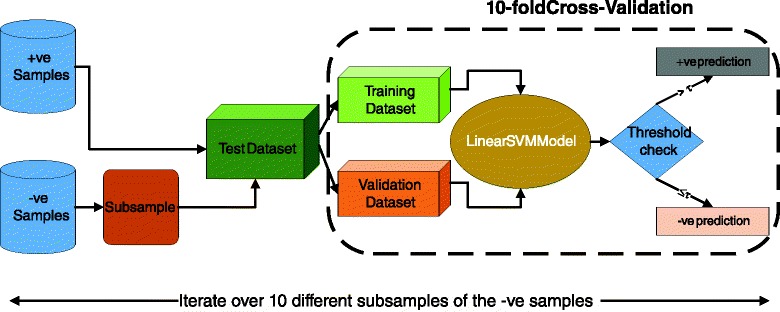
Workflow of the validation protocol used to evaluate *Avishkar*. There are 100 different true positive rate and false positive rate values (10 from the sub-samples of the -ve samples times 10 from the cross-validation) that are averaged to create the ROC curves

We show the overall workflow for the validation protocol in Fig. 10. The results shown in Fig. 3 were generated by sub-sampling from the set of negative (larger) miRNA-mRNA interactions in order to have roughly the same number of positive and negative examples in each iteration. Then, for each iteration, we used 10-fold cross-validation to evaluate the performance of our model on the hold-out (validation) dataset. For each run, we computed the true-positive and false-positive rates by varying the threshold for the probability scores generated by our model. We then averaged the true-positive rate and the false-positive rate, obtained over the 100 hold out datasets (10 sub-sampling runs and 10-fold cross-validation for each run). For inter-species validation, we similarly sub-sampled from both the human dataset and the mouse dataset, in order to have roughly the same number of positive and negative examples in each iteration. However, instead of doing cross-validation, we trained on the human dataset and used the mouse dataset as test and vice versa. We then averaged the true-positive rate across the 10 validation datasets, obtained by sub-sampling, for both the human and mouse datasets.

We downloadeded target locations and their corresponding scores for each mRNA, as computed by mirSVR [26], PITA [32], TargetScan [33], and STarMir [27]. When comparing performance against competition we only considered those miRNA-mRNA pairs for which we generated data. Then, we labeled each miRNA-mRNA interaction, reported by other methods, as 1 or 0, depending on whether the reported target location was contained within an AGO cross-linked region for the mRNA in the CLIP-seq datasets. Since, mirSVR reports sequences for hg19 assembly of the human genome, while we generated predictions using the hg18 assembly, we mapped mRNA target sites from hg19 assembly to the hg18 assembly. Then, we computed mean ROC (receiver operating characteristics) curves, for each method from the scores and the computed CLIP labels (1/0).

We also evaluated the performance of our method against another method—MIRZA [28], on the human dataset. The biophysical model developed in [28] also considers all possible canonical and non-canonical seed matches to identify miRNA target sites. We downloaded the MIRZA tool from their website [69]. To generate ROC curves for MIRZA, we ran MIRZA on our candidate set of positive and negative examples. Since MIRZA requires that all target sites be of the same length, we made sure that each target site was expanded, or shrunk if necessary, to have a length of 50 nucleotides. We averaged the ROC curve for MIRZA over 5 runs, where in each run, we randomly sub-sampled negative examples to have, roughly, the same number of positive and negative examples. The ROC curve for each run was generated by varying the threshold for the target quality score computed by MIRZA to compute the true- and false-positive rates.

## Endnotes

^1^ Throughout this paper, we will synonymously and interchangeably use the terms “non-canonical match”, “seedless match”, and “non-canonical seedless match”.

^2^*Avishkar* means “discovery” in Sanskrit. The word captures our enthusiasm in using functional data analysis techniques to extend and refine the discovery of genomic targets modulated by these small, albeit powerful regulatory RNA—miRNA, which can chisel the process of gene regulation, post-transcriptionally. This in turn will accelerate the discovery of novel disease biomarkers [70, 71] that can cause network perturbations, *in vivo* [72], and facilitate the development of novel miRNA-based therapeutics [73].

^3^ However, this discussion of seedless matches has to be balanced with the fact that the level of downregulation of gene expression is higher for seed matches [28].

^4^ Loosely speaking, “alignment score” is a quantitative value that represents how well the miRNA is paired with the mRNA. So the score depends on the lengths of exact matches and the degree of mismatches.

^5^ It should be noted that our definition of a seed match is slightly different from what others have used in the past. We used a slightly more general definition of a canonical seed match to account for different types of canonical seed matches that are considered by various computational methods. For example, Bartel *et al.* [4] define three types of alignments involving perfect complementarity with nucleotides 2–7 from the 5’ end of the miRNA as canonical seed match.

^6^ For TargetScan[33] we reduced the threshold to 80 % since a threshold of 90 % resulted in 0 overlaps with CLIP-seq data.

^7^ For seed matches, the sparse 5’ UTR match sites show slightly better performance, but considering the small size of this sample set, this is likely not statistically significant.

^8^ For seed sites, there are not enough positive examples for the 5’ UTR to draw statistically significant conclusions.

## Abbreviations

AGO: Argonaute
API: Application program interface
AUC: Area under the curve
CDS: Coding sequence
CLIP: Crosslinking immunoprecipitation
FPR: False positive rate
HEK: Human embryonic kidney
MRE: miRNA recognition element
NCBI: National Center for Biotechnology Information
REST: Representational state transfer
RISC: RNA-induced silencing complex
ROC: Receiver operating characteristics
SVM: Support vector machines
SVR: Support vector regression
TPR: True positive rate
UTR: Untranslated region
