# Erratum to: ‘MicroRNA target prediction using thermodynamic and sequence curves’

**DOI:** 10.1186/s12864-016-2367-1

**Published:** 2016-03-09

**Authors:** Asish Ghoshal, Raghavendran Shankar, Saurabh Bagchi, Ananth Grama, Somali Chaterji

**Affiliations:** Department of Computer Science, Purdue University, West Lafayette, IN 47907 USA

Unfortunately, the original version of this article [1] contained an error. Figures 6 and 7 were incorrect with the wrong captions. The figures have been corrected in the original article and is also included correctly below.

**Fig. 6:**
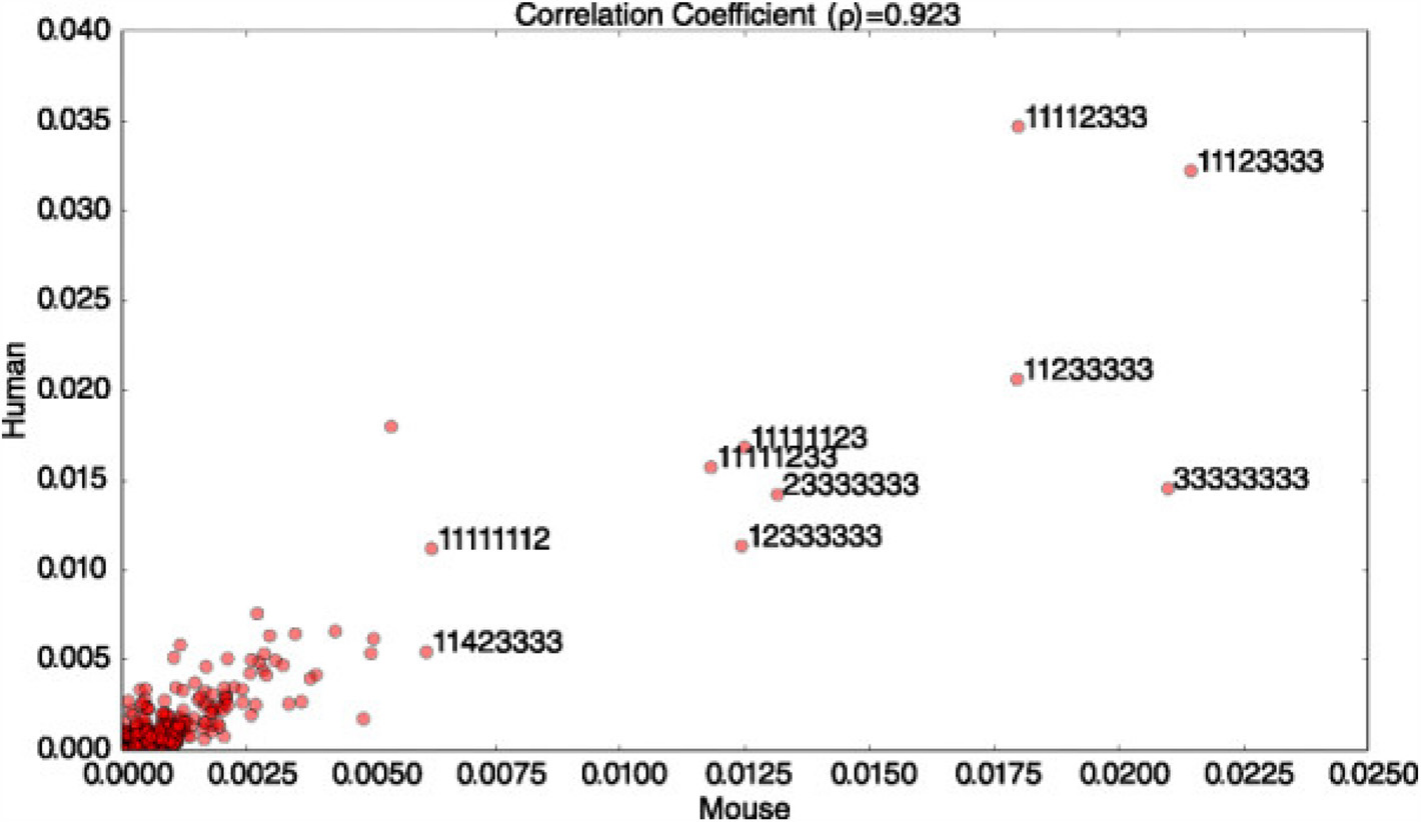
Scatter plot of frequencies of various types of seed alignment patterns in set of positive miRNA-mRNA interactions for Mouse (x-axis) and Human (y-axis). Among the top-10 most frequently occurring patterns, only two, namely, the 6-mer and 7-mer, are canonical seed match patterns. In the labels for the top-10 most frequently occurring patterns, 1 indicates a match, 2 indicates a mismatch, 3 a gap, and 4 indicates a GU wobble

**Fig. 7:**
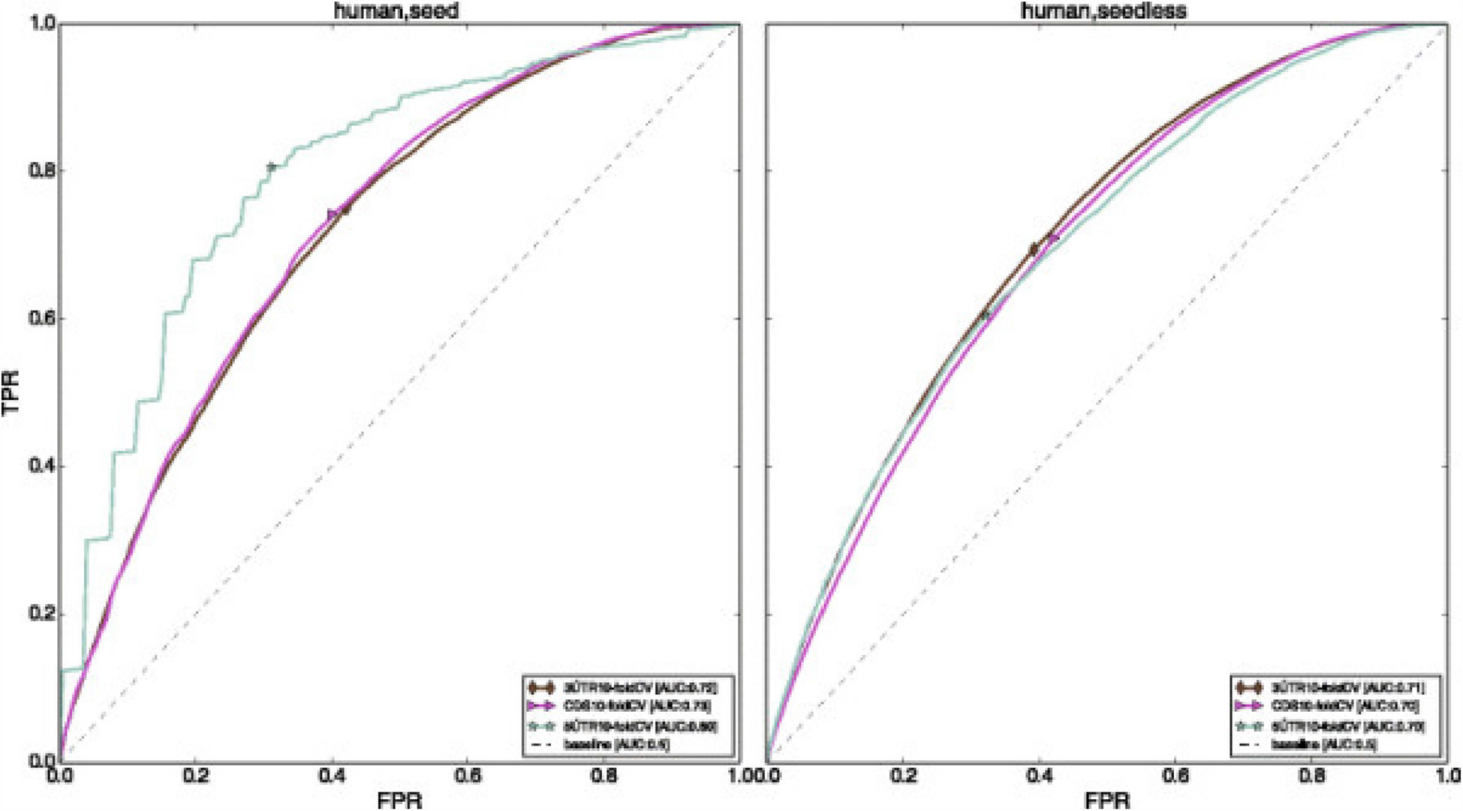
ROC curves for 10-fold cross-validation performance of *Avishkar* in different regions of the gene in the human dataset
